# MutComFocal: an integrative approach to identifying recurrent and focal genomic alterations in tumor samples

**DOI:** 10.1186/1752-0509-7-25

**Published:** 2013-03-25

**Authors:** Vladimir Trifonov, Laura Pasqualucci, Riccardo Dalla Favera, Raul Rabadan

**Affiliations:** 1 Department of Biomedical Informatics, New York, NY, 10032, USA; 2 Center for Computational Biology and Bioinformatics, New York, NY, 10032, USA; 3 Institute for Cancer Genetics, Herbert Irving Comprehensive Cancer Center, Columbia University, New York, NY, 10032, USA; 4 Department of Pathology and Cell Biology, Columbia University, New York, NY, 10032, USA; 5 Department of Genetics and Development, and Department of Microbiology and Immunology, Columbia University, New York, NY, 10032, USA

**Keywords:** Tumorigenic mutations, Driver genes, Data integration

## Abstract

**Background:**

Most tumors are the result of accumulated genomic alterations in somatic cells. The emerging spectrum of alterations in tumors is complex and the identification of relevant genes and pathways remains a challenge. Furthermore, key cancer genes are usually found amplified or deleted in chromosomal regions containing many other genes. Point mutations, on the other hand, provide exquisite information about amino acid changes that could be implicated in the oncogenic process. Current large-scale genomic projects provide high throughput genomic data in a large number of well-characterized tumor samples.

**Methods:**

We define a Bayesian approach designed to identify candidate cancer genes by integrating copy number and point mutation information. Our method exploits the concept that small and recurrent alterations in tumors are more informative in the search for cancer genes. Thus, the algorithm (Mutations with Common Focal Alterations, or MutComFocal) seeks focal copy number alterations and recurrent point mutations within high throughput data from large panels of tumor samples.

**Results:**

We apply MutComFocal to Diffuse Large B-cell Lymphoma (DLBCL) data from four different high throughput studies, totaling 78 samples assessed for copy number alterations by single nucleotide polymorphism (SNP) array analysis and 65 samples assayed for protein changing point mutations by whole exome/whole transcriptome sequencing. In addition to recapitulating known alterations, MutComFocal identifies ARID1B, ROBO2 and MRS1 as candidate tumor suppressors and KLHL6, IL31 and LRP1 as putative oncogenes in DLBCL.

**Conclusions:**

We present a Bayesian approach for the identification of candidate cancer genes by integrating data collected in large number of cancer patients, across different studies. When trained on a well-studied dataset, MutComFocal is able to identify most of the reported characterized alterations. The application of MutComFocal to large-scale cancer data provides the opportunity to pinpoint the key functional genomic alterations in tumors.

## Background

Most cancers occur as a result of changes in the genome of somatic cells. While such changes often have no effect, some alterations in combination with the environment of the cells that harbor them, conspire to initiate and maintain a tumorigenic process resulting in abnormal cell proliferation. Discovering the genomic lesions contributing to tumor initiation and progression will provide a more profound understanding of the biology of cancer, new prognostic markers, and hopefully novel therapeutic targets. Our improved understanding of cancer genetics is the result of the revolution in experimental tools available to make observations at different dimensions and scales. Large scale projects, such as The Cancer Genome Atlas (TCGA) or the International Cancer Genome Consortium (ICGC) [1] aim to provide a comprehensive characterization of nearly 50 tumor types. These projects collect large numbers of tumor samples, providing information on clinical and high throughput biological data, including mRNA and small RNA expression, point mutations, methylation, and copy number copy number (CN) alterations. Recently, many studies have reported recurrent genetic alterations in a variety of tumors by analyzing high throughput data in large panels of tumor samples (e.g. [2]–[6]).

Although we can observe a cancer genome at nucleotide resolution with high throughput sequencing, the impact of particular alterations is not clear. The ultimate answer can only be obtained from a detailed functional analysis of the biological consequences of the mutations. Unfortunately, this is a laborious process and the complexity of the mutational landscape makes impossible to assess the impact of all reported alterations. Thus it is desirable to prioritize the mutations identified with sequencing analysis in order to guide the subsequent functional validation. One can heuristically predict the impact of gene mutations, such as nonsense mutations and frameshift indels, on the structure of the protein product using indicators of selection pressure such as dn/ds test. Alternatively, one can rely on the statistical likelihood of mutational recurrence across many different samples. It is expected that driver genes with a significant contribution to the etiology of a particular cancer type will be altered repeatedly across many different samples in proportion significantly higher than passenger mutations.

Several approaches have been proposed to identify the most relevant recurrent alterations across a large number of tumor samples. GISTIC [7] (Genomic Identification of Significant Targets in Cancer) aims to identify significant chromosomal aberrations in tumors by studying alterations in large collections of copy number data. The method identifies genomic regions that present more aberrations in tumors than random. Other groups [8,9] have proposed to combine copy number data with expression data to pinpoint driver genes within regions of frequent chromosome aberrations. In [5] we proposed a heuristic measure of focality as a way of identifying focal alterations in copy number data. A problem with copy number analysis is that often the genomic alterations are not sufficiently focal to identify the genes that are the target of a particular genomic lesion, as opposed to those alterations that are there by chance only. A direct solution to the lack of specificity in copy number analysis is provided by high throughput sequencing technologies, which have been the object of many exciting developments in the last decade, providing a read-out of the cancer genome at nucleotide level.

In this work we propose a framework, MutComFocal, for assessing the importance of genes using sequencing and copy number data from multiple samples of the same cancer type. The method formalizes in a Bayesian framework a previous heuristic idea [5] used to identify focal copy number aberrations. The measure proposed here takes into account the recurrence of altered genes as well as the size of the lesions. Genes are scored separately for deletions and gains, with the assumption that genes with high CN gain score will tend to be oncogenes, while those with high deletion score will tend to be tumor suppressors. In this way, focality of the lesions altering a particular region is not inferred entirely from the recurrence of alterations to genes in that region, but includes also the size of the lesions covering it. As a test of the applicability of the method we use several datasets from studies with non-overlapping samples of DLBCL. In each case, the method confirms the genes that were previously reported as contributing to the pathogenesis of the disease and identifies some new targets.

## Results

### Focality and recurrence from copy number data and mutations

**Figure 1:**
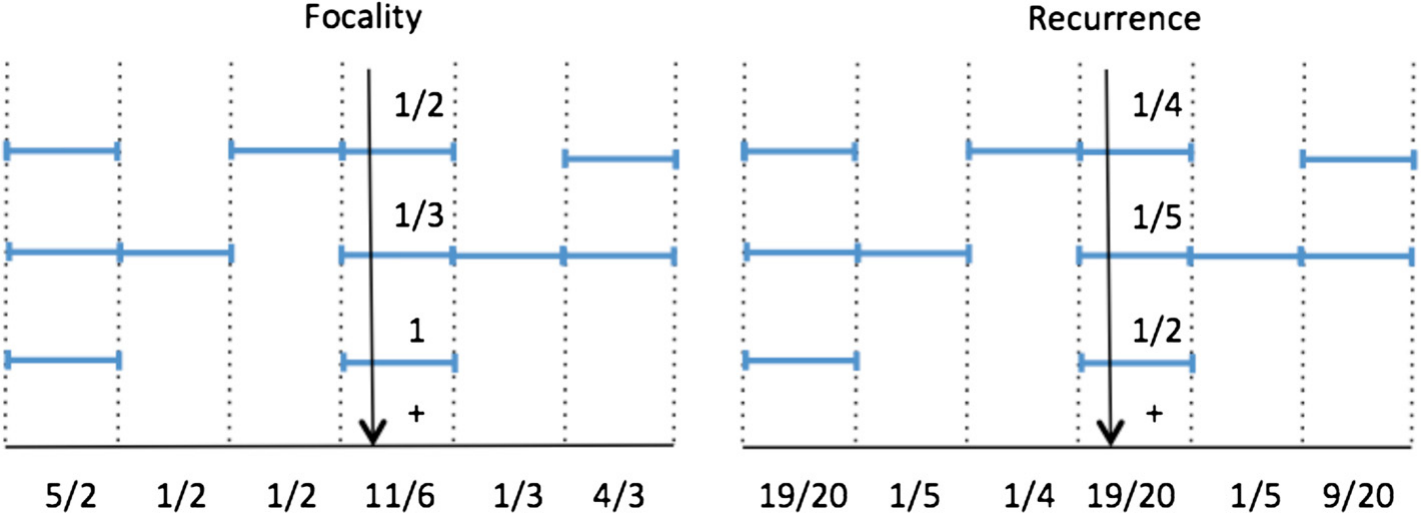
**Focality and recurrence scores measure the presence of a given gene in alterations in tumor samples.** The focality score (left) assigns equal weight to all genes participating in a genomic alteration inversely proportional to the size of that alteration, while recurrence score (right) assigns equal weight to all genes altered in a sample inversely proportional to the total number of gene altered in the sample. In the figures, the alterations occurring in a particular sample are represented by segmented horizontal lines and each segment is a particular gene. For example, the top sample has three alterations with one, two, and one genes, respectively.

The method is based on two measures of the significance of each gene (see Figure 1): a focality score and a recurrence score. Both scores are computed separately for CN gains and deletions. First, for a fixed sample each of the measures assigns a score to every gene. The focality score of a gene in a fixed sample is *D/L*, where *D* is the amplitude (deviation from normal of the copy number, 2 for a diploid chromosome), and *L* is the length of that lesion, in terms of the number of genes contained. For example, in an autosomal chromosome a loss of two copies has deviation *D* = 2, a loss of one copy has *D* = 1, a gain of one copy has *D* = 1, etc. The recurrence score of a gene in a sample is *D/T*, where *D* is the amplitude of the lesion covering the gene and *T* is the sum of the deviations of the genes altered in the sample. Next, the scores for each gene are summed across the samples and normalized across the genes to sum to 1. The focality and recurrence scores are combined into a single score by multiplication and then normalization to 1. The combined score forms a distribution over the genes and, as explained in the methods, genes are divided into tiers using the entropy function. The scores are formally introduced within the Bayesian framework in the Methods section. The main quantity to be computed is the probability that a gene is a driver given a set of alterations.

While CNVs provide information on genomic lesions spanning several genes, point mutations provide more specific information on single genes. To integrate mutations, we devise a method that considers both mutations and copy number alterations within the same framework (MutComFocal). We first compute a mutation score in a manner analogous to computing the recurrence score by considering mutated genes to participate in single gene lesions. More precisely, in a fixed sample which has *N* mutated genes, each mutated gene receives a sample mutation score of *1/N*. The mutation scores are obtained by adding sample mutation scores over all samples followed by normalization to 1. The mutation scores are combined separately with recurrence and focality scores for CN gains and deletions to obtain the gain/mutation and deletion/mutation scores for each gene. More precisely, for every gene, the gain/mutation score is equal to the product of the mutation score and the sum of the recurrence and focality gain scores for that gene. Similarly, using recurrence and focality deletion scores, we obtain deletion/mutation scores. The gain/mutation and deletion/mutation scores are normalized to sum to 1.

### Results in DLBCL data

We performed the MutComFocal analysis for identifying driver genes in diffuse large B-cell lymphoma using copy-number data from 78 DLBCL samples (dbGaP Study Accession: phs000328.v1.p1 [5]) and somatic mutations data from 65 cases, including 49 samples from Lohr et al. [3] corresponding to 3351 mutated genes, 6 samples from Pasqualucci et al. (89 mutated genes) [5], and 10 samples from the Morin et al. study [10]; from the latter study, only mutations obtained by DNA sequencing and confirmed to be somatic were selected (in total, 183 mutated genes). Keeping only genes recurrently mutated resulted in 712 genes. Genes were scored according to their copy number status from SNP array analysis as explained in the methods section.

**Figure 2:**
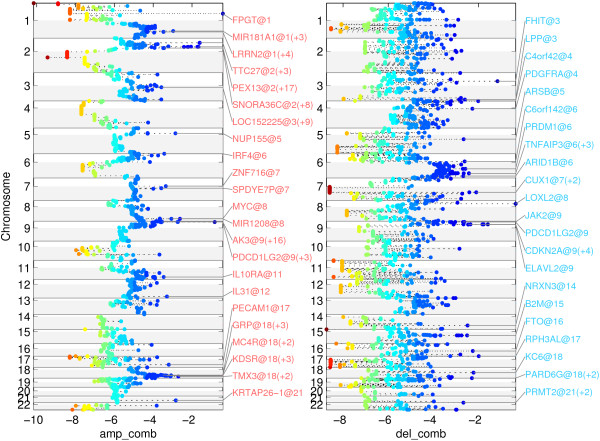
Representation of the MutComFocal focality score for gains (left) and deletions (right). Leader genes, i.e. the genes with highest score in each region, and their chromosomal location are indicated in the figure.

**Figure 3:**
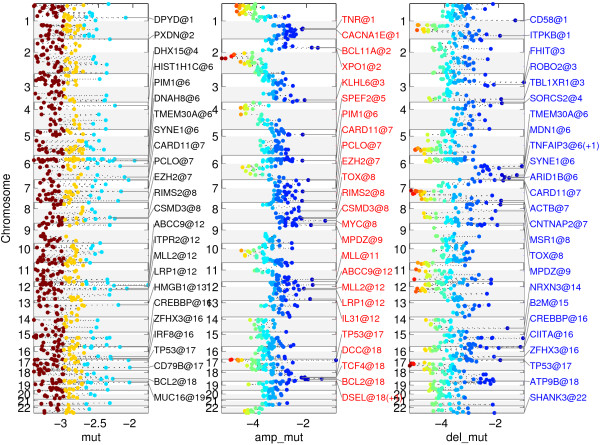
Representation of the MutComFocal score for mutations (left) integrated with gains (center) and deletions (right).

Figure 2 represents the scores for the 22,636 genes of the human genome on the autosomal chromosomes annotated with the names of the leaders of the top 25 regions. For each region, the gene with the highest score is declared a leader of the region (for a full description of the genes included in each of the regions refer to Additional file 1: Table S1 and Additional file 2: Table S2). Adding the mutation data to the copy number analysis data resulted in the scores shown in Figure 3, where we have listed the top 25 genes (for a full description of the genes included in each of the regions refer to Additional file 3: Table S3 and Additional file 4: Table S4).

The leader genes in the top deleted regions contained genes with a known contribution to the pathogenesis of DLBCL, such as CDKN2A , B2M [11], PRDM1 [12], TNFAIP3 [13], and NRXN3, a gene previously noted to be altered in DLBCL [5]. The top genes also included some known fragile sites, as FHIT in 3p14.2 (FRA3B, [14]) and IMMP2L in 7q31.1-7q31.2 (FRA7G, [15]). In addition, the method identified the PDCD1LG2/CD274 region [16,17], 17p13.3, a region that has been implicated as a tumor-suppressor region in medulloblastoma, where it is deleted in 40% of cases [18] and ARID1B, a member of the SWI/SNF chromatin remodeler complex implicated in differentiation and development [19]. ARID1B is deleted in 24% of cases and mutated in 6% of cases.

Adding the information on somatic mutations to the copy number analysis confirmed several genes obtained from copy number data only, such as B2M, NRXN3, FHIT, TNFAIP3, and ARID1B. In addition, this analysis brought forth genes that have been recorded previously to contribute to DLBCL such as CIITA [20], CREBBP [4], CD58 [11] and TP53 [5], as well as genes that have been associated to DLBCL such as TMEM30A, ACTB, ITPKB and TBL1XR1 [3,5]. The analysis also singled out ROBO2 (deleted in 9% of cases and mutated in 3%) a candidate tumor suppressor in head and neck cancer [21], and MSR1 (deleted in 22% of cases and mutated in 3%), a gene with tumor suppressor function in leukemia stem cells of chronic myeloid leukemia [22]. Surprisingly, CARD11 also scored high by this analysis. This observation can be attributed to the fact that the analysis does not distinguish deleterious mutations from those that confer an activating effect.

A similar analysis of CN gains produced regions encompassing known contributors to DLBCL such as PDCDLG2/CD274, BCL2, and MYC, all of which are targeted by amplifications. The appearance of PDCDLG2/CD274 as a relevant target of both CN gains and losses is an indication of a more complex behavior of the region, potentially induced by a genomic translocation [20]. The method also identified a region containing REL [23] and a region containing NFKBIZ, which have been singled out previously as occurring frequently in DLBCL and studied in that context [24]. In addition, a region containing MDM4, a gene frequently observed to be overexpressed in human tumors and contributing to tumor formation in mice by inhibiting TP53 [25], was identified. Among the top 25 genes obtained by integrating CN gains with somatic mutations were known DLBCL-associated oncogenes such as PIM1 [26], CARD11 [27], MYC, EZH2 [28], and BCL2 [29]. In addition, the analysis identified KLHL6 (a target of CN gains in 23% of cases and mutations in 8%), a gene involved in BCR signaling and germinal center formation in mice [30], IL31 (CN gains in 18% of cases and mutations in 3%), a gene involved in the activation of JAK/STAT, PI3K/AKT and MAPK signaling [31], and LRP1 (gained in 24% of cases and mutated in 3%), a gene promoting cancer cell invasion [32].

We compared the performance of MutComFocal with the result produced by GISTIC, a widely used tool for analyzing copy number data. The results of GISTIC in the DLBCL data are shown in the Supplementary Information (Additional file 5: Figure S1, Additional file 6: Figure S2, in Additional file 7: Table S5, Additional file 8: Table S6, and Additional file 9: Table S7). GISTIC produces two levels of q-values – one at the level of probes (given in Additional file 7: Table S5**)** and a second one at the level of peaks, which are contiguous genomic regions spanning one or more genes (given in Additional file 8: Table S6 and Additional file 9: Table S7). We compared the analysis of MutComFocal to both levels of q-values produced by GISTIC. To this end, for a particular level of q-value we sorted the genes by that q-value and selected the top 25 regions in the manner described above (results in Additional file 10: Table S8, Additional file 11: Table S9, Additional file 12: Table S10, and Additional file 13: Table S11). For probe level q-values, the top 25 regions of gain included 1336 genes and the top 25 deletion regions contained 1225 genes; 7 of the top 25 regions of gain produced by GISTIC appeared also in the top 25 regions produced by MutComFocal (namely, those containing REL, DSEL/BCL2, PTPRC/MDM4, PDCD1LG2/CD274, AK3, IL10RA, and SPDYE7P) (Additional file 14: Table S12); 8 of the top 25 deletions regions produced by GISTIC, containing CDKN2A, B2M, TNFAIP3, PRDM1, ARID1B, FHIT, RPH3AL, LOXL2, MTAP, and, C4orf42, overlapped with the results of MutComFocal (Additional file 15: Table S13). For peak level q-values, the 15 regions of gain contained 950 genes (Additional file 8: Table S6) and the 14 deletion regions contained 223 genes (Additional file 9: Table S7); 7 of the 15 amplification peak regions produced by GISTIC, containing REL, BCL2, PDCD1LG2, IL10RA, NFKBIZ, MYC, and NUP155, overlapped with MutComFocal (result in Additional file 16: Table S14); 4 of the 14 deletion peak regions produced by GISTIC, containing CDKN2A, PRDM1, TNFAIP3, and FHIT, overlapped with MutComFocal (result in Additional file 17: Table S15). In summary, the comparison of the two methods shows that at a high level they capture similar recurrent alterations. In addition, by incorporating somatic mutations MutComFocal is able to narrow down those regions to specific genes, thus reducing significantly the number of candidate genes implicated in cancer.

### Discussion and conclusions

Novel driving alterations in cancer provide the opportunity for the discovery of potential molecular targets in cancer, and the identification of prognostic and diagnostic alterations. With the advent of novel high throughput technologies in biomedical research, several comprehensive molecular characterization initiatives have been put forward. At least three large consortiums, The Cancer Genome Atlas (TCGA), The International Cancer Genome Consortium (ICGC) and the NCI's Therapeutically Applicable Research to Generate Effective Treatments (TARGET) are collecting samples in hundreds of patients. As diverse high throughput data from large scale projects become available, there is a growing need of methods to (1) integrate and (2) extract the biologically relevant information. In this paper, we present a Bayesian framework aiming to identify candidate genes implicated in oncogenesis combining copy number and point mutation data. MutComFocal is a method based on the concept that small focal alterations are more informative than large chromosomal aberrations. Compared to methods based only on recurrent copy number alterations, MutComFocal is able to pinpoint candidate driver genes in regions encompassing large genomic alterations.

We have applied MutComFocal to CNV and point mutation data in DLBCL. Apart from previously reported genes, MutComFocal has identified several potential novel genes that may be involved in DLBCL development: ARID1B, ROBO2 and MRS1 as candidate tumor suppressors and KLHL6, IL31 and LRP1 as candidate oncogenes. Among them, ARID1B is a member of the SWI/SNF chromatin remodeler complex and has been implicated both in transcriptional activation and repression by chromatin remodeling [15]. It is found deleted in 24.4% of the samples and mutated in 6.2%. Other members of the SWI/SNF complex are also found mutated (ARID1A with inactivating mutations in 4.1% cases) and deleted (SMARCA4 deleted in 10.3% of cases) suggesting the possible involvement of the SWI/SNF complex in DLBCL.

The application of MutComFocal to other tumor types is straightforward. MutComFocal provides not only a method for integrating somatic point mutations and copy number alterations, but a general Bayesian framework that can be easily extended to other types of data.

## Methods

Given a genome, successive lesions altering the function of the genome’s component genes may eventually result in expression of a novel phenotype, e.g. cancer. In this work, lesions can be copy number gains/amplifications, deletions, or mutations. Eventually the genome accumulates several lesions and expresses the novel phenotype if one of them includes a driver gene. We equate the genotype resulting from a sequence of lesions with the genes altered by those lesions. We observe the genotypes of genomes that are known to express the novel phenotype and our goal is to recover the genes driving the genotype’s selection. We define two scores to measure the importance of a given gene: a focality and a recurrence score. The focality score is local in nature and depends on the sizes of the lesions which contain the gene, while the recurrence score is global in nature and depends on the total number of genes altered in a sample. The formal description is formulated within the Bayesian framework, and the two scores are in fact the two posterior distributions for the likelihood that a particular gene drives the phenotype, derived correspondingly from local and global priors for that event. The details are given below.

### Mutation

In the MutComFocal framework, a genomic lesion is represented by the set of genes altered by that lesion. A lesion *M* is a result of a random process with a given distribution *P*^*m*^*(M)*, which specifies the probability of occurrence of the lesion *M* due to *m*utation. Let *S* = {*M*_1_, ⋯, *M*_*t*_} be a set of disjoint lesions representing all the lesions occurring in a particular sample. The genotype U *S* resulting from the set *S* consists of all genes appearing in the lesions in the set *S*, i.e. US=UiMi. The set *S* is distributed according to a given distribution *P*^*m*^*(S)*, which specifies the probability of occurrence of the lesions in the set *S* due to *m*utation. The exact shapes of the mutation distributions for lesions and sets of lesions are not relevant for this work and will not be discussed further.

### Selection

Consider lesions of a fixed type: gain/amplification, deletions, or mutations. To model selection we assume that the genotype resulting from a disjoint set of such lesions *S* = {*M*_1_, ⋯, *M*_*t*_} expresses the phenotype when a lesion in the sequence contains a driver gene. We consider two possibilities for the posterior probability that a given gene *D* is a driver of the phenotype, given that the genotype of the set *S* expresses the phenotype: a global posterior and a local posterior. To obtain the two posteriors we define first the likelihood that a lesion *M* is a driver lesion given that *D* is a driver gene as

$$L\left(M|D\right)\;\underline{\underline{\mathrm{deff}}}\;P^{m}\left(M\right)\cdot \delta \left(D\in M\right),$$

where *δ(D ∈ M)* is 1 if the lesion *M* includes the gene *D* and 0 otherwise, with corresponding posterior

$$P\left(D|M\right)=\frac{\delta \left(D\in M\right)\cdot P^{d}\left(D\right)}{\sum_{G\in M}P^{d}\left(G\right)},$$

where *P*^*d*^*(D)* is the prior probability that *D* is a *d*river of the phenotype. We similarly have the likelihood that *S* expresses the phenotype, assuming that lesion *M* is a driver, and the corresponding posterior as

$$\begin{array}{ll} L\left(S|M\right) & \underline{\underline{\mathrm{deff}}}\;P^{m}\left(S\right)\cdot \delta \left(M\in S\right)\;\mathrm{and}\\ P\left(M|S\right) & =\frac{\delta \left(M\in S\right)\cdot P^{d}\left(M\right)}{\sum_{i}P^{d}\left(M_{i}\right)}, \end{array}$$

where *P*^*d*^*(M)* is the prior that *M* is a driver lesion and δ(M ∈ S) is 1 if the set *S* includes the lesion *M* and 0 otherwise. The posterior that the gene *D* is a driver gene, assuming that the genotype of the set *S* expresses the phenotype, is defined as $P\left(D|S\right){\;}^{\underline{\underline{\mathrm{deff}}}}\;\sum_{i}P\left(D|M_{i}\right)\cdot P\left(M_{i}|S\right)$. To obtain the global posterior *P*^*g*^(*D*|*S*) we assume that *P*^*d*^(*M*) ∝ ∑ _*G* ∈ *M*_*P*^*d*^(*G*) in which case

$$P^{g}\left(D|S\right)=\frac{\delta \left(D\in \;U\;S\right)\cdot P^{d}\left(D\right)}{\sum_{G\in \;U\;S}P^{d}\left(G\right)}\;\underline{\underline{\mathrm{deff}}}\;R\left(D|S\right),$$

For the local posterior *P*^*l*^(*D*|*S*)  we assume that *P*^*d*^ (M) ∝ 1 and obtain

$$P^{l}\left(D|S\right)=\underset{i}{\sum }\frac{\delta \left(D\in M_{i}\right)\cdot P^{d}\left(D\right)}{t\cdot \sum_{G\in M_{i}}P^{d}\left(G\right)}\;\underline{\underline{\mathrm{deff}}}\;F\left(D|S\right)$$

We refer to the global posterior *R*(*D*|*S*)  as a *recurrence score* and to the local posterior *F*(*D*|*S*)  as a *focality score.*

Assume a uniform distribution for the prior probability *P*^*d*^*(D)* that gene *D* is a driver. If the gene *D* does not appear in the genotype of the set *S*, then *R*(*D*|*S*) = *F*(*D*|*S*) = 0. Otherwise, if *K* is the number of genes in the genotype of the set *S* and the gene *D* appears in a lesion of size *L*, then *R*(*D*|*S*) = 1/*K* and *F*(*D*|*S*) = 1/(*t* · *L*). Thus, under the global posterior, the genes from a set of lesions are assigned the same probability of being a driver, independent of the size of the lesions they belong to. Under the local posterior, on the other hand, all lesions have an equal chance of containing a driver, but once a lesion is fixed its genes are equally likely to drive the phenotype. Hence, recurrence scores all genes in a genotype equally while focality scores the genes depending on the size of the lesions they belong to, so that genes from smaller lesions score higher.

Recurrence and focality scores are computed separately for each of the three types of lesions. Note that for mutations, the recurrence and focality scores are equal, because every mutation affects a single gene. Thus for every gene in the sample we have 5 scores: *R*^*amp*^, *F*^*amp*^, *R*^*del*^, *F*^*del*^, and *R*^*mut*^.

We extend the above ideas to incorporate various copy numbers for deletions/amplification or many mutations per gene for mutations by modeling lesions as a pair *M =(C,N)* where *C* is a positive real number and *N* is a set of genes. In the case of deletions/amplifications, *C* is the number of copies lost/gained by the lesion. We modify the recurrence score so that $P^{d}\left(M\right)\propto C\cdot \underset{G\in N}{\sum }P^{d}\left(G\right)\;$ and the focality score so that *P*^*d*^(*M*) ∝ *C*. This way we obtain

$$\begin{array}{ll} R\left(D|S\right) & \;\underline{\underline{\mathrm{deff}}}\;\frac{\sum_{i}C_{i}\cdot \delta \left(D\in N_{i}\right)\cdot P^{d}\left(D\right)}{\sum_{i}C_{i}\cdot \sum_{G\in N_{i}}P^{d}\left(G\right)}\;\mathrm{and}\\ F\left(D|S\right) & \underline{\underline{\mathrm{deff}}}\;\underset{i}{\sum }\frac{C_{i}}{\sum_{j}C_{j}}\cdot \frac{\delta \left(D\in N_{i}\right)\cdot P^{d}\left(D\right)}{\sum_{G\in N_{i}}P^{d}\left(G\right)}. \end{array}$$

### Genotype data from several samples

So far our discussion has focused on the lesions observed in a single sample, and we have defined the recurrence and focality scores as two posterior distributions over the genes. To combine genotype data from several samples, we obtain an empirical prior P_∞_*(D)*, defined below, suitable to the observed samples.

Consider the observation that a prior distribution *P*_*0*_*(D)* and the likelihood probability *P*(*S*|*D*) imply a posterior $P_{0}^{p}\left(D|S\right)$, from which we can obtain a distribution *P*_*1*_*(D)* by summing over *S* in the following way:

$$P_{1}\left(D\right)\;\underline{\underline{\mathrm{deff}}}\underset{S}{\sum }P_{0}^{p}\left(D|S\right)\cdot P\left(S\right),$$

where *P(S)* is a distribution from which the samples were generated. Iterating this process we obtain distributions *P*_*i*_(*D*), *i* = 1, 2, which converge to a fixed point *P*_*∞*_(*D*). Since

$$P\left(S\right)=\underset{D}{\sum }P\left(S|D\right)\cdot P_{\infty }\left(D\right),$$

the prior P_∞_*(D)* has the property that together with the likelihood function it predicts the data perfectly.

In practice, obtaining P_∞_*(D)* by the above iterative procedure is prevented by two obstacles: first, the distribution *P (S)* is not available and, second, iterating to infinity is not viable. To overcome the first obstacle, we substitute the iteration with its empirical analogue

$$P_{j+1}\left(D\right)\;\underline{\underline{\mathrm{deff}}}\;\frac{1}{T}\underset{i}{\sum }P_{j}^{p}\left(D|S_{i}\right),$$

where *S*_1_, ⋯, *S*_*T*_ are the observed samples. The second obstacle is resolved by repeating the above process a sufficiently high number of times rather than to infinity. Thus, as a stopping criterion we use the Kullback–Leibler divergence as a measure of distance between two consecutive members of the sequence of distributions and stop as soon as the divergence is less than a fixed amount.

### Defining gene tiers

A posterior distribution *P (D)* over the genes obtained by the methods described above implies an ordering of the genes such that the genes with highest posterior probability receive our highest confidence to be drivers of the phenotype. For the purpose of incorporating this ordering into further analyses it is beneficial to discretize it into a small number of tiers. To achieve this we use the entropy *H (P)* of the distribution

$$H\left(P\right)=-\underset{D}{\sum }\;P\left(D\right)\cdot \log_{2}\;P\left(D\right)$$

Let *P*_*1*_*= P* and declare all genes which have $P_{1}\left(D\right)>2^{-H\left(P_{1}\right)}$ to be in the 1^st^ tier. Next we define the distribution *P*_*2*_ (*D*) to be zero for the genes which were declared to be in the 1^st^ tier and equal to *P*_*1*_*(D)* for rest, and normalize it to sum to 1. We use *P*_*2*_*(D)* and its entropy to define the 2^nd^ tier genes. This process is repeated until there are no more genes to consider.

This procedure is motivated by the property of entropy to be 0 iff *P*_*1*_*(D)* is focused on a single gene and log_2_*N* iff *P*_*1*_*(D)* is uniform over the genes (*N* is the total number of genes). Thus, intuitively, we can think of a distribution with entropy *h* as being uniform on *2*^*h*^ genes. This intuitive statement is made more precise by noting that for any distribution *P (D)* and ε > 0, by Markov’s inequality in probability theory, we have that

$$\underset{P\left(D\right)\leq 2^{-\left(1+\varepsilon \right)∙H\left(P\right)}}{\sum }P\left(D\right)\leq \frac{1}{1+\varepsilon }.$$

Hence genes with *P*(*D*) > 2^- (1 + *ε*) · *H*(*P*)^, of which there can be at most 2^(1 + *ε*) · *H*(*P*)^, contain at least 1–1/ (1+*ε*) of the weight of the distribution *P (D)*.

## Competing interests

The authors declare that they have no competing interests.

## Authors’ contributions

LP and RDF have provided the seed idea behind focally as evidence of significance and help in the biological and clinical interpretation of findings. RR and VT implemented and developed the idea. All authors read and approved the final manuscript.

## Supplementary Material

Additional file 1 ComFocal: top 25 amplified regions. Click here for file

Additional file 2 ComFocal: top 25 deleted regions. Click here for file

Additional file 3 MutComFocal: top 25 amplification/mutation regions. Click here for file

Additional file 4 MutComFocal: top 25 deletion/mutation regions. Click here for file

Additional file 5 GISTIC amplifications. Click here for file

Additional file 6 GISTIC deletions. Click here for file

Additional file 7 GISTIC probe scores. Click here for file

Additional file 8 GISTIC peak amplifications scores. Click here for file

Additional file 9 GISTIC peak deletions scores. Click here for file

Additional file 10 GISTIC: top 25 probe level amplification regions. Click here for file

Additional file 11 GISTIC: top 25 peak level amplification regions. Click here for file

Additional file 12 GISTIC: top 25 probe level deletion regions. Click here for file

Additional file 13 GISTIC: top 25 peak level deletion regions. Click here for file

Additional file 14 GISTIC/ComFocal comparison for probe level amplifications. Click here for file

Additional file 15 GISTIC/ComFocal comparison for probe level deletions. Click here for file

Additional file 16 GISTIC/ComFocal comparison for peak level amplifications. Click here for file

Additional file 17 GISTIC/ComFocal comparison for peak level deletions. Click here for file
