# Supplementary figures and images for: MutComFocal: an integrative approach to identifying recurrent and focal genomic alterations in tumor samples
Source: BMC Syst Biol. 2013 Mar 25;7:25. doi: 10.1186/1752-0509-7-25 (PMC3637169; doi:10.1186/1752-0509-7-25)

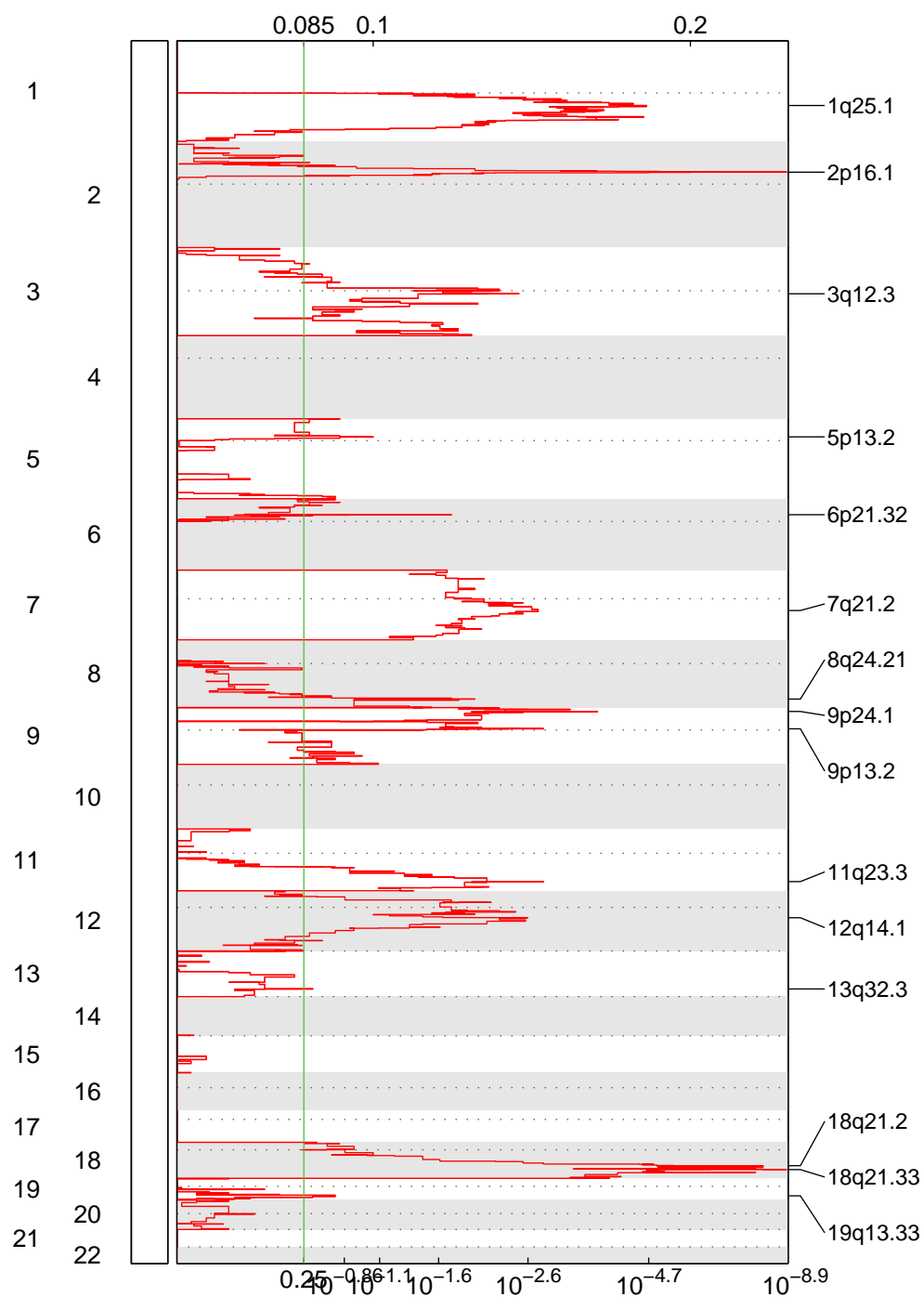

Supplement: Additional file 5 — GISTIC amplifications. [file 1752-0509-7-25-S5.pdf]

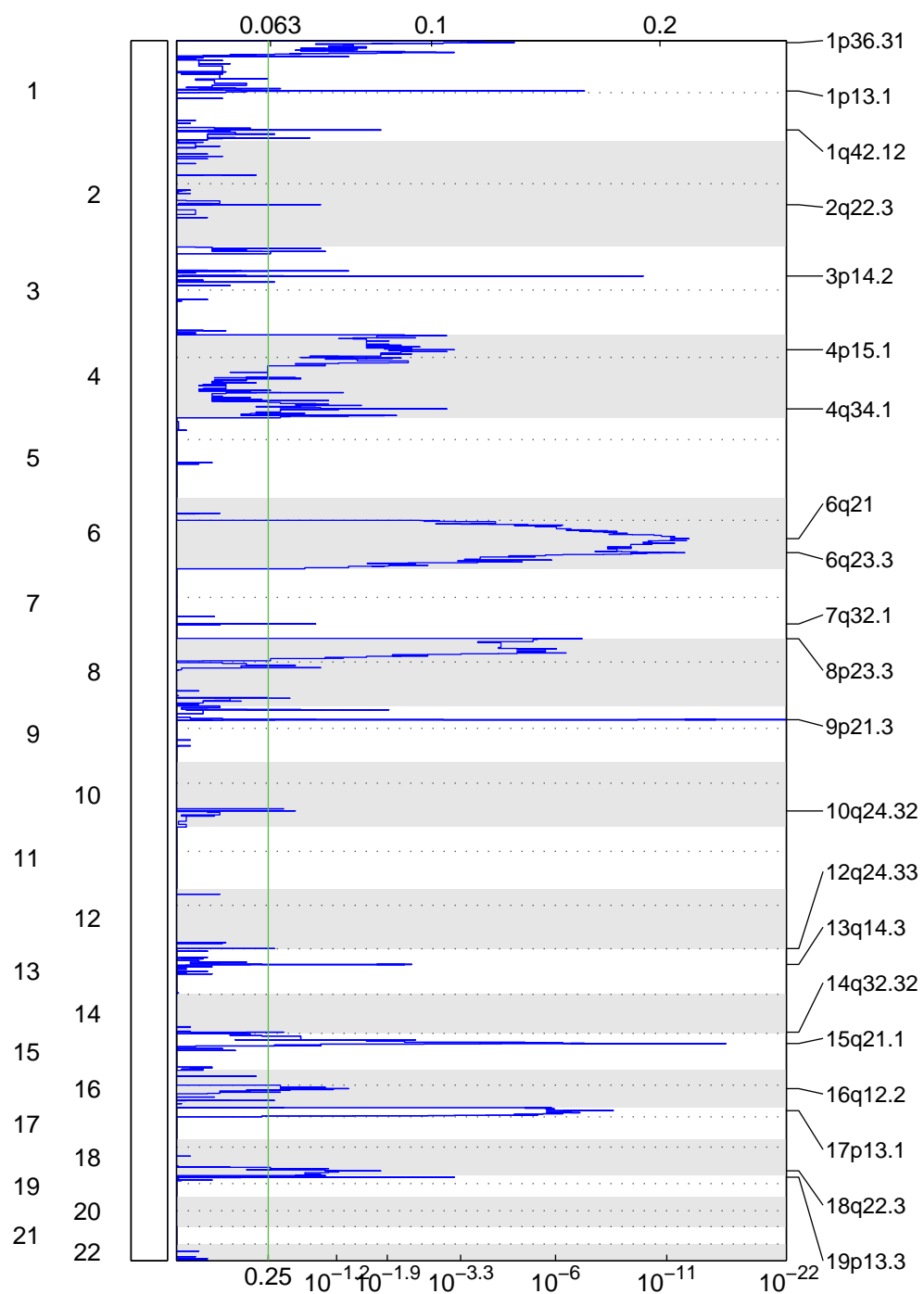

Supplement: Additional file 6 — GISTIC deletions. [file 1752-0509-7-25-S6.pdf]
